# Design and Characterization of a Silk-Fibroin-Based Drug Delivery Platform Using Naproxen as a Model Drug

**DOI:** 10.1155/2012/490514

**Published:** 2012-02-27

**Authors:** Tatyana Dyakonov, Chue Hue Yang, Derek Bush, Saujanya Gosangari, Shingai Majuru, Aqeel Fatmi

**Affiliations:** Banner Pharmacaps Inc., 4125 Premier Drive, High Point, NC 27265, USA

## Abstract

The objective of this proof-of-concept study was to develop a platform for controlled drug delivery based on silk fibroin (SF) and to explore the feasibility of using SF in oral drug delivery. The SF-containing matrixes were prepared via spray-drying and film casting, and the release profile of the model drug naproxen sodium was evaluated. Attenuated total reflectance Fourier transform infrared spectroscopy (FTIR) has been used to observe conformational changes in SF- and drug-containing compositions. SF-based films, spray-dried microparticles, and matrixes loaded with naproxen were prepared. Both FTIR spectra and *in vitro* dissolution data demonstrated that SF **β**-sheet conformation regulates the release profile of naproxen. The controlled release characteristics of the SF-containing compositions were evaluated as a function of SF concentration, temperature, and exposure to dehydrating solvents. The results suggest that SF may be an attractive polymer for use in controlled drug delivery systems.

## 1. Introduction

Silk fibroin (SF) is a natural polymer produced by a variety of insects and spiders. The best characterized silks are the dragline silk from the spider *Nephila clavipes* and the cocoon silk from the domesticated silkworm *Bombyx mori*, which has been used in textile production clinical sutures, and more recently as a scaffold for tissue regeneration [1–3]. *Bombyx mori *silk is composed of a filament core protein, silk fibroin, and a glue-like coating consisting of a nonfilamentous protein, sericin. SF is characterized by repetitive hydrophobic and hydrophilic peptide sequences [4] and consists of heavy and light chain polypeptides of ~390 kDa and ~26 kDa, respectively, linked by a disulfide bond at the C-terminus of the two subunits. The primary structure of *Bombyx mori* SF protein is characterized by the presence of three amino acids in a roughly 3 : 2 : 1 ratio: glycine (45%), alanine (30%), and serine (12%); and the sequence is dominated by [Gly-Ala-Gly-Ala-Gly-Ser]_n_. SF chains also contain amino acids with bulky and polar side chains, in particular tyrosine, valine, and acidic amino acids [5]. The repetitive sequence in hydrophobic residues dominates the **β**-sheet structure, forming crystalline regions in SF fibers and films. The formation of these **β**-sheets results in insolubility in water. Hydrophobic regions of silk fibroin in aqueous solution assemble physically by hydrophobic interactions and eventually organize into hydrogels.

Silk fibroin exhibits impressive mechanical properties as well as biocompatibility making it an attractive biomaterial and scaffold for tissue engineering. The fibroin protein is one kind of biological materials used for artificial skin and other medical applications. As a result of its biodegradability [6], SF was evaluated for several biomedical applications. In one example [7], SF-based films with a thickness of 10–100 *μ*m were developed for acceleration of wound healing and could be peeled off without damaging the newly formed skin. As such, the application of wound protective membranes made from SF was investigated [8]. SF is considered a suitable material for skeletal tissue engineering because of its good oxygen and water-vapor permeability and its minimal inflammatory reaction *in vivo *[6, 9]. As reported previously [10], fibroin hydrogel scaffolds were prepared from SF aqueous solution with addition of 30% glycerol to promote *in situ* bone regeneration. Also, SF was investigated as the substratum for the culture of animal cells in place of collagen [11]. In another application, the aqueous SF solution was used to prepare a membrane for immobilization of *Aspergillus niger*, glucose-oxidase, and *Pseudomonas fluorescens* lyophilized cells [12]. A novel biocompatible blend [13] was prepared from recombinant human-like collagen (RHLC) and used as a scaffold material for hepatic tissue engineering applications. Solution blending was used to incorporate RHLC with SF to enhance the blend films biocompatibility and hydrophilicity, while maintaining elasticity. In yet another demonstration of SF utility, three-dimensional microperiodic scaffolds for tissue engineering were produced from aqueous solutions of regenerated *Bombyx mori* silk [14]. The scaffolds supported human bone-marrow-derived mesenchymal stem cell (hMSC) adhesion and growth.

Sericin and fibroin have been recently explored in the field of drug delivery. SF was studied as an organic polymer for controlled drug delivery [4], in which dextrans of different molecular weights, as well as proteins, were physically entrapped into the drug delivery device during processing into films. The release behavior of benfotiamine, a vitamin B1 derivative, from SF gel was investigated [15]. Microspheres were fabricated from an aqueous SF solution by laminar jet break-up flow and were investigated as a platform for controlled drug delivery [16]. The assembly process was reported for SF particles loaded with small molecule model drugs, such as alcian blue, rhodamine B, and crystal violet, produced by an all-aqueous salting out process [17], and it was demonstrated that the release kinetics of crystal violet is dependent on the secondary structure of the SF particles.

We attempted to design an oral drug delivery system based on the ability of SF to undergo conformational transition from a random coil to a *β*-sheet form to induce crystallinity and produce an interpenetrating network (IPN). Several different approaches to develop a SF-based drug delivery system were used: (1) film and matrix casting with varying composition of SF, gelatin, glycerin and the model drug, and (2) spray drying of SF/model drug solution.

 Multiple factors were evaluated for their effect on SF *β*-sheet formation, including solvents, SF molecular weight, silk source, and so forth. The aim of our study is also to understand the silk fibroin processing and control of structure in connection with design of a controlled release matrix.

## 2. Materials and Methods

### 2.1. Reagents and Chemicals

Cocoons of *Bombyx mori* silkworm silk were kindly provided by M. Goldsmith (University of Rhode Island, USA). Low MW (~14 kDa) SF powder was supplied by Lalilab (Raleigh, USA). Raw silk fiber (Grade 5A, *Bombyx mori *silk) was purchased from RIA International LLC (East Hanover, NJ, USA), and Fibro-Silk Powder (MW ~ 100 kDa) was purchased from Arch Chemicals, Inc (Atlanta, GA, USA). Both Sephadex G-25 (medium grade) and sodium carbonate were purchased from J. T. Baker (Austin, TX, USA). Naproxen sodium was supplied by RoChem International, Inc (Ronkonkoma, NY, USA). Sodium dodecyl sulfate and calcium chloride dihydrate were purchased from Spectrum Chemical (New Brunswick, NJ, USA). Lithium bromide, calcium nitrate, and potassium bromide were purchased from Sigma-Aldrich (St. Louis, MO, USA). Gelatin (Type B, 150 Bloom limed bone, NF) was obtained from Rousselot (France). Glycerin (USP, Kosher, vegetable-based) was obtained from Proctor and Gamble (Cincinnati, OH, USA). All other chemicals were of analytical or pharmaceutical grade, were purchased from Sigma-Aldrich, and were used without any additional purification.

### 2.2. Silk Blend Preparation

Silk fibroin aqueous stock solutions were prepared as described previously with some modifications [16, 18]. Briefly, cocoons, silk powder, or grade 5A raw silk were boiled several times for 1 hour in aqueous solutions of 0.02 M Na_2_CO_3_, or 0.25% NaCO_3_/0.25% NaSO_4_ mixture, rinsed thoroughly with distilled water to remove the glue-like sericin proteins and dried. Dry degummed silk fibers were then dissolved in one of the following neutral solutions of LiBr, Ca(NO_3_)_2_ or CaCl_2_, and then the resulting solution was dialyzed against distilled water using a Slide-A-Lyzer dialysis cassette (MWCO 3500, Pierce) or cellulose membrane tube (MWCO 6000–8000, Sigma-Aldrich) at room temperature to remove the salt. The completion of the dialysis process was monitored by conductivity measurement. Undissolved particles were removed by centrifugation. The final concentration of SF aqueous solution was determined by weighing the residual solid of a known volume of solution after drying at 60°C for 2 days. Based on this determination, the concentration of the silk protein was approximately in the range of 3 to 4% (w/v). To prepare films, SF solution was transferred to a polystyrene weighing boat and allowed to dry for several days at room temperature in a desiccator. SF/gelatin films were prepared by mixing the SF solution with gelatin blends, consisting of gelatin, plasticizer, and water, and dried in a polystyrene weighing boat at room temperature in a desiccator for several days.

### 2.3. Purification of Silk Solution by Column Chromatography Using Sephadex G-25

Separation of salts and SF protein was performed using a Sephadex G-25 media column as described in the literature [19] with some modifications. SF powder was dissolved in a triad solvent of CaCl_2_ : EtOH : H_2_O with a mole ratio of 1 : 2 : 8, at a concentration of 14.4% (w/w), at 60–80°C, and stirred for 4–6 hrs until fully dissolved and the stock SF solution was diluted in deionized water to reduce sample viscosity. To a 7.3 g of Sephadex G-25 (medium grade) 42.6 g water was added allowing the Sephadex to swell for at least 3 hours then the slurry was packed by gravity flow of deionized water (2-3 bed volumes) in a 50 mL glass burette. Conductivity of eluent flow was measured until 3 consecutive fractions (10 mL each) tested <10 *μ*S/cm to ensure removal of contaminating ions from column before addition of SF solution (7.2% SF). Fractions were collected every 5–10 minutes for the first ~25 minutes, while conductivity was continuously measured then every 2–5 minutes until the end of the experiment, or until the conductivity of the eluting fraction returned to a value of <10 *μ*S/cm. UV absorbance was measured and recorded for each fraction at 280 nm (blank : quartz cuvette filled with deionized water). All fractions were placed in the oven at 60°C for 24 hours, or until all liquid had evaporated, and the residual net mass was determined for each fraction after drying.

### 2.4. Preparation of SF Microparticles

To prepare SF microparticles, the model drug naproxen sodium (NS), was dissolved in SF solution (silk : naproxen ratios tested: 1 : 1, 1.5 : 1, 2 : 1, and 3 : 1) for spray-drying. Naproxen-sodium-containing SF microparticles were prepared using a bench top spray-dryer (BÜCHI B-290 model, Switzerland). The adjustable parameters included inlet and outlet temperature, solution pump flow rate, and the aspirator partial vacuum. In the present experiments, the inlet temperature ranged from 135 to 200°C, the pump flow rate was 6-7 mL/min, and the atomizing air flow rate was 600 l/h (0.75 bar).

### 2.5. Scanning Electron Microscopy

The morphological characteristics of SF microparticles and cross-sections of Gelatin/SF blends, both before and after treatment with dehydrating solvents and before and after drug release experiments, were examined by scanning electron microscopy (SEM). Samples of dried films were prepared for SEM imaging by flash freezing in liquid nitrogen and immediately fracturing with a sharp blade to produce cross-sections. The samples were then coated with a ~8 nm layer of Au/Pt mist using a sputter coater. A Hitachi S3200 variable pressure scanning electron microscope equipped with an E-T secondary electron detector was used to examine the samples at an accelerating voltage of 5 kV and a working distance of 15 mm.

### 2.6. Dynamic Light Scattering

Particle size measurements for silk microparticles were also performed using dynamic light scattering (DLS). A Nicomp ZW380 (Particle Sizing systems, Inc. Ca) particle sizer at fixed angle (90°) and 632.8 nm was employed for size distribution measurements. The SF microparticles were suspended in an oil matrix at a 1 : 100 dilution before measurements.

### 2.7. Infrared Spectroscopy

FTIR spectra of silk fibroin blend films were collected using a Nicolet 510P spectrometer equipped with the attenuated total reflectance (ATR) accessory. These films were cast on the surface of polystyrene weigh boats directly from solution. Each spectrum for samples was acquired in transmittance mode with a spectral range of 4000–400 cm^−1^. FTIR spectra of the SF-containing microparticles were collected using a Perkin-Elmer Spectrum BX FTIR spectrophotometer and NIC380 Avatar OMNI Sampler apparatus. KBr pellets, containing ~0.7% (w/w) of spray-dried powder were prepared using a Carver Pellet Press with 18,000 lbs applied load. Thin films for comparison were obtained by casting of SF solution on the surface of polystyrene weigh boats and were analyzed by FTIR.

### 2.8. In Vitro Drug Release Studies

The naproxen release from the films, matrixes, and spray-dried microparticles was studied using Distek Evolution 6100 dissolution system. Two sets of conditions were used: one-stage method with apparatus 2 (paddle) at 50 rpm in 500 mL of pH 7.4 phosphate buffer at 37°C and three-stage method with three pH buffers—simulated gastric fluid (pH 1.2), simulated intestinal fluid (pH 4.8) and pH 7.4 buffer. The samples (1.5 mL) at each predetermined time point were analyzed by HPLC according to naproxen sodium, USP method. The detector wavelength was set at 272 nm and the injection volume was 10 *μ*L. The HPLC column used was Inertsil ODS-3 4.6 × 250 mm, 5 *μ*m with the column temperature maintained at 25°C. The mobile phase was a binary mixture of potassium phosphate buffer : acetonitrile (3 : 2) at pH 3.0 pumped at flow rate of 1.5 mL/min.

## 3. Results

### 3.1. Silk Fibroin Processing

Raw silk consists of fibroin that is bound together by hydrophilic gum-like protein, the sericin. For use in biomedical applications, silk protein fibroin has to be dissolved. The dissolution of SF as related to the roles of salts, alcohol, and water and coagulation of the fibroin solution was discussed elsewhere [20]. *Bombyx mori* SF dissolves in neutral salt-alcohol systems without degradation. Lithium bromide—or lithium thiocyanate—ethanol system, hexafluoroisopropyl alcohol, and calcium nitrate-methanol systems have been widely used to dissolve silk fibroin. A summary of the processing conditions employed for different sources of SF in our research is presented in Table 1.

**Figure 1:**
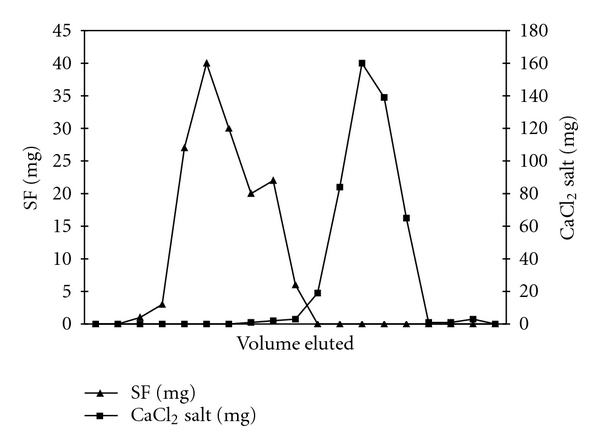
Purification of SF solution (7.2%) by Sephadex G-25 (medium grade) column chromatography using gravity flow. Elution of SF (–▲–) and CaCl_2_ (–■–) salt is shown as net dry mass recovered in each fraction collected, and both are confirmed by UV absorbance at 280 nm and conductivity measurements, respectively.

It usually takes longer time (7-8 hours) at 65–80°C to dissolve raw *Bombyx mori* silk using different solvent systems (Table 1), then to dissolve SF powder (4–6 hours). In order to develop a scalable process we have attempted to separate SF from salts by Sephadex G-25 media as described in the literature [19]. The primary purpose of purifying by column chromatography was to explore the feasibility of a quicker processing step, in place of dialysis. The two components were effectively separated using this approach, allowing for gravimetric analysis as a means of approximating the mass of either SF protein or CaCl_2_ salt contained in each fraction collected (Figure 1). These results demonstrate the feasibility of separating SF protein dissolved in a highly concentrated salt solution; however, further characterization of the SF protein after desalting will be required prior to implementation of this process on a commercial scale.

In order to reduce the processing time we explored the applicability of different sources of silk fibroin. One option was to eliminate the degumming step and use partially hydrolyzed SF, while another option was to use a low-molecular-weight water-soluble SF, thus allowing elimination of three processing steps: degumming, dissolving, and dialysis. The ability to form crystalline structure was investigated for partially hydrolyzed SF at three different molecular weights (100 kDa, 14 kDa, and 2 kDa). Low MW SF (14 and 2 kDa) was soluble in water and demonstrated crystalline secondary structure in pure form as evidenced by FTIR data presented in Table 2. However, only higher-molecular-weight SF (100 kDa) was shown to possess *β*-sheet conformation in blends with gelatin.

### 3.2. Effect of Different Solvents on *β*-Sheet Formation

The effect of glycerin and dehydrating solvents (methanol, ethanol, and isopropyl alcohol) on formation of **β**-sheets was studied for gelatin/silk fibroin compositions. SF/gelatin compositions with and without glycerin were prepared and treated with methanol, ethanol, and isopropyl alcohol. The effect of different solvents and presence of glycerin on **β**-sheet formation is summarized in Table 3. The effect of solvents on **β**-sheet formation showed that films treated with different alcohols exhibit the signature of the **β**-sheet conformation (silk II structure) at each amide peak: amide I, which reflects the stretching of C=O group along the SF backbone (shifted from ~1650 cm^−1^ to 1630 cm^−1^). The amide II, which originates from N–H deformation, shifts from ~1544 to 1536 cm^−1^. As seen from Table 3, isopropyl alcohol treatment also gives good crystallizing effect.

The content of SF and glycerin has an impact on the FTIR signal. Higher SF (SF/G 1 : 1) content showed higher fibroin-specific signal change for amide I after cast-treatment. Interestingly, the untreated films (SF/G 1 : 1) also demonstrated the crystallizing effect indicating that glycerin could induce the formation of **β**-sheet (Table 3).

### 3.3. Preparation of Drug-Loaded Films

A mixture of dialyzed SF solution with predetermined amount of gelatin mass and a model drug was cast on a polystyrene weighing boat to prepare SF films. Cast films were treated with methanol, ethanol, and isopropyl alcohol or exposed to water vapor. Gelatin mass was prepared from gelatin, water, and plasticizer (glycerin) by initially mixing water and plasticizer with gelatin granules followed by heating at ~60°C until a clear gel was obtained.

### 3.4. Development of a Sustained Release Matrix

SF-containing compositions were prepared, using naproxen sodium, as a model drug and presented in Table 4. These compositions are calculated based on weight after the films and matrixes have completely dried, before performing dissolution testing.

**Figure 2:**
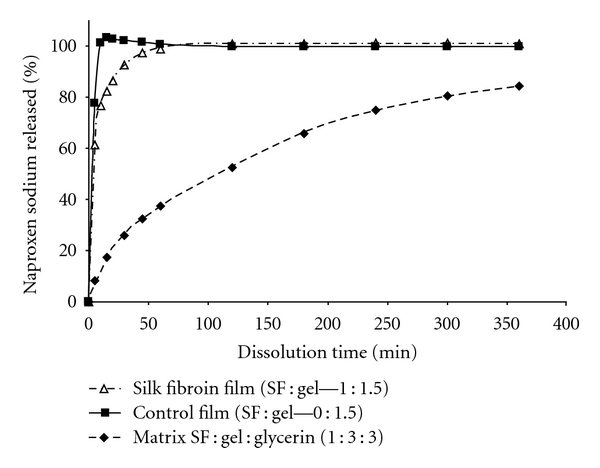
Dissolution profile of naproxen from SF-containing matrix (♦) as compared to SF (*▵*) and non-SF (■) film.

The characterization of naproxen release from SF-containing matrixes and films was performed at pH 7.4. Drug release from amorphous carrier (control film) was characterized by an initial burst exceeding 75% of the theoretical amount of naproxen in 5 minutes demonstrating immediate release of the model drug. For SF-containing films the initial burst was markedly reduced (~60% in 5 minutes). Studies (Figure 2) indicated that the time needed to achieve over 80% dissolution for naproxen-loaded films is 15 minutes as opposed to 5 hours for the SF-containing matrix. These results demonstrate the formation of crystalline SF network in silk and gelatin blends which significantly retard the release of naproxen compared to amorphous gelatin.

### 3.5. Development of SF Microparticles for Controlled Release

Although the SF/gelatin/glycerin blends described above demonstrated feasibility for use as a controlled drug delivery system, another approach utilizing microparticles containing only SF and water was explored. SF-containing compositions loaded with naproxen sodium were prepared via spray-drying and are presented in Table 5.

**Figure 3:**
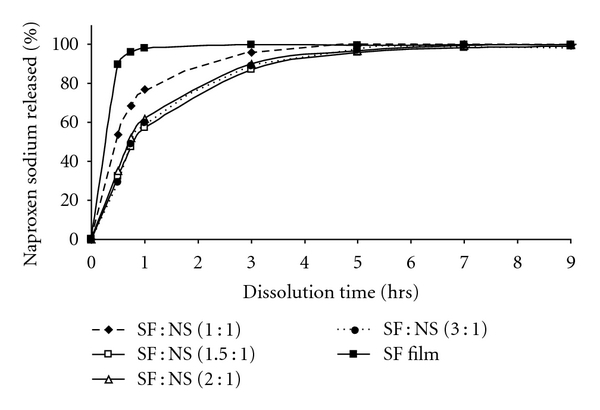
One-stage dissolution profile (pH = 7.4): comparison of SF : NS spray-dried powders; SF : NS 1 : 1 (♦), SF : NS 1.5 : 1 (□), SF : NS 2 : 1 (*▵*), SF : NS 3 : 1 (*⚫*), and naproxen-loaded SF film (■).

The spray-dried powders with different ratios of SF : NS (1 : 1, 1.5 : 1, 2 : 1 and 3 : 1) were encapsulated in 2-piece hard gelatin capsules, and the release of naproxen sodium was studied at pH 7.4. The dissolution study results were compared to the release profile of naproxen-loaded SF/gelatin thin films. As shown in Figure 3 SF films released 90% of naproxen sodium within 1 hour of dissolution time at pH 7.4, while SF spray-dried powder released only ~50% of naproxen sodium. Approximately 4 hours were required for SF spray-dried powder to release above 80% of naproxen sodium. The controlled release observed for naproxen from the microparticles compared to the fast release rate from the film suggests that the spray-drying process induces a unique change to the structure of the fibroin microparticles thereby transforming them into prospective sustained release vehicles. This provided a starting point for developing SF spray-dried microparticles as a drug delivery system.

**Figure 4:**
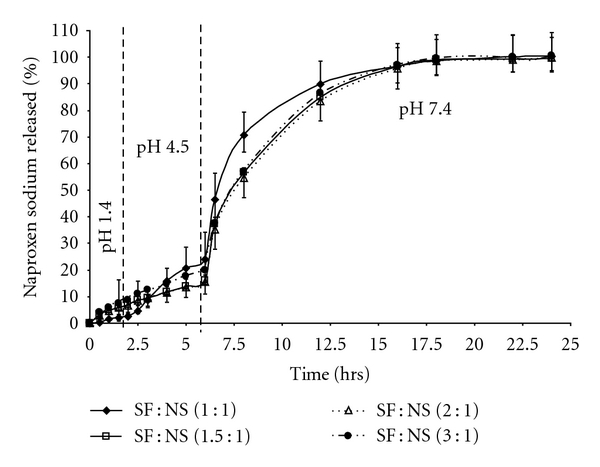
Naproxen release profiles from SF : NS spray-dried microparticles: three-stage dissolution at pH 1.4, 4.5 and 7.4: comparison of SF : NS spray-dried powders; SF : NS 1 : 1 (♦), SF : NS 1.5 : 1 (□), SF : NS 2 : 1 (*▵*), and SF : NS 3 : 1 (*⚫*).

The release of naproxen sodium from spray-dried microparticles was also demonstrated using a three-stage dissolution method at pH 1.4, 4.5 and 7.4. The microparticles with SF:NS ratios of 1.5 : 1, 2 : 1, and 3 : 1 were observed to have similar release profiles while microparticles with SF : NS ratio of 1 : 1 had slightly higher release profiles. At stage 3 (pH 7.4) dissolution, microparticles with different SF : NS ratios performed similarly, releasing up to 80% NS after 3 hours (Figure 4), while at stage 2 (pH 4.5) the release of the drug was in the range of 10–20%. 

As seen in Figure 4, <25% of drug release from SF microspheres is observed over the first 6 hours at or below pH 4.5, even at the lowest ratio of SF : NS (1 : 1). Complete drug release is only observed after 15 hours at pH 7.4 (25 hours total dissolution time) for all samples analyzed.

FTIR analysis was performed on spray-dried microparticles, and the data showed that spray-drying of SF solution induced *β*-sheet conformation which was indicated by the amide I band shifting from 1650 cm^−1^ to 1642/1631 cm^−1^ and amide II from 1536 cm^−1^ to 1516 cm^−1^. However, most of the analyzed spray-dried microparticles did not show *β*-sheet transition. Samples exposed to 76% relative humidity for one week showed *β*-sheet transition as evidenced by the shift of amide I, II, and III bands (Table 6), thus demonstrating that exposure to humidity induced *β*-sheet formation.

To further investigate the effect of water vapor treatment on conformation change we subjected the SF : NS spray-dry powder exposed to 76% relative humidity to 3-stage dissolution studies. The slight changes in dissolution may suggests that exposure to moisture contributed to conformational transition in SF spray-dried powder. SF : NS (2 : 1) spray dried powder exposed to 76% relative humidity provides similar release during stage 1 and 2 dissolution testing, but showed slightly lower NS release during stage 3 testing. These data could support the hypothesis that the conformational transition rate depended on the rearrangement of hydrogen bonds between SF chains; however additional studies are needed for confirm this assumption. Previous work has shown that the conformation transition of SF from random coil to *β*-sheet is due to the rearrangement of the hydrogen bonds between the polypeptide chains and the transition may be a nucleation-dependent aggregation [21].

**Figure 5:**
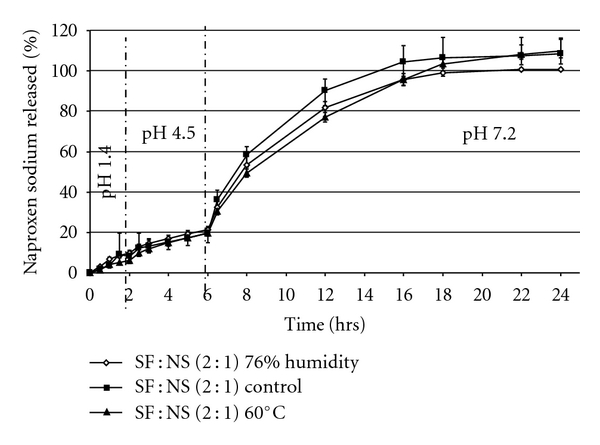
Comparison of naproxen release from SF : NS (2 : 1) spray-dry powder; non-exposed (■) to 76% relative humidity (Control), exposed (*◊*) to 76% relative humidity and accelerated stability (▲) at 60°C.

The modest effect on dissolution performance observed (Figure 5) was due to the fact that when both the SF microparticles that were initially exposed to water and those not exposed to water are subjected to the dissolution media, they can further rearrange their chains making the initial humidity effect negligible.

Additional accelerated stability studies on naproxen-containing SF microparticles were performed. The data presented in Figure 5 demonstrated that the release profile of naproxen from SF microparticles exposed to either high humidity (76%) or 60°C for 1 week is comparable to naproxen release from control samples.

**Figure 6:**
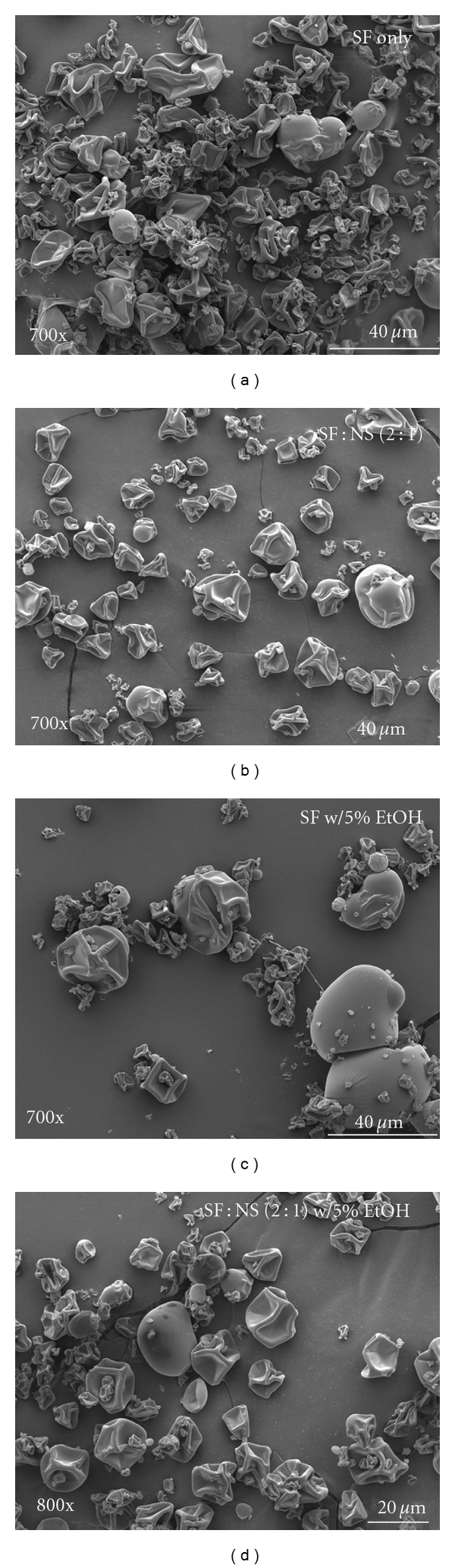
SEM images of spray-dried microparticles. SF solution with spray-dried microparticles (a), SF : NS (2 : 1) blend spray-dried microparticles (b), SF solution with additional 5% ethanol spray dried microparticles (c), and SF : NS (2 : 1) blend with additional 5% ethanol spray dried microparticles (d).

SEM images of SF microparticles obtained by spray drying are illustrated in Figure 6. SEM Images 6(a), 6(b), 6(c), and 6(d) show the microparticles formed from each spray-dried powder blend. The images provide evidence that microparticles were formed during the spray drying process. As seen in the images the SF microparticles collapsed, leaving behind imploded micro-particulates. The addition of 5% ethanol to microparticles loaded with naproxen and control blends (refered to as (c) and (d) in Figure 6) was shown to produce microparticles that are less aggregated and more smooth, on average, which is possibly attributed to an accelerated evaporation process in the presence of alcohol. Further studies of spray-drying conditions are required to address the observation of collapsed SF microparticles. The volume, intensity, and number weighted analysis with the DLS instrument showed that the mean particle size for the SF : NS (2 : 1) microparticles was 26.05 ± 1.92 *μ*m. This is in close agreement with the particle size established by the SEM images. 

**Figure 7:**
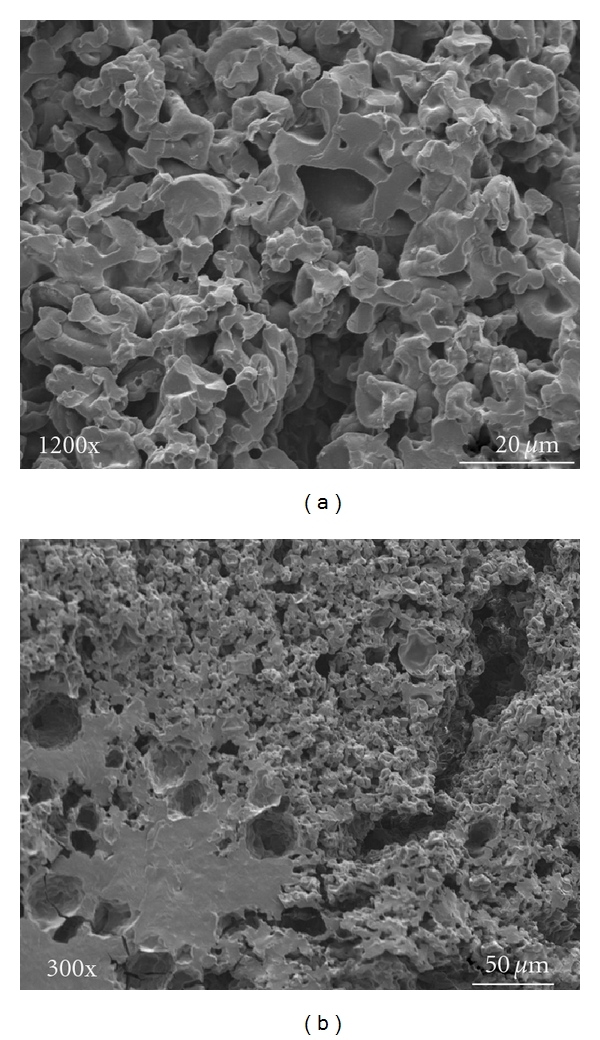
SEM images obtained from different spray-dried microparticles extracted with different dissolution media after *in vitro* release study. SF : NS (2 : 1) porous matrix remaining from macroparticles after dissolution ((a) and (b)).

A series of SEM images (Figure 7) were acquired for different spray-dried microparticles remaining after 3-stage dissolution experiments at pH 1.4, 4.5 and 7.4. The microparticles were retrieved from the dissolution vessel after the endpoint at pH 7.4 and dried before SEM analysis. SEM Images 7(a), and 7(b) represent dried SF samples that remained as a compact porous matrix.

## 4. Discussion

### 4.1. Silk Fibroin Processing

Natural silk fibers dissolve only in a limited number of solvents, compared to globular proteins, because of the presence in fibroin of a large amount of intra- and intermolecular hydrogen bonds and its high crystallinity and specific physicochemical properties. The isoelectric point of fibroin varies in the range pH 3.6–5.2, depending on the conditions of solution preparation [22]. Fibroin dissolves in concentrated aqueous solutions of acids (phosphoric, formic, sulfuric, and hydrochloric) and in concentrated aqueous, organic, and aqueous-organic solutions of salts [LiCNS, LiBr, CaCl_2_, Ca(CNS)_2_, ZnCl_2_, NH_4_CNS, CuSO_4_ + NH_4_OH, Ca(NO_3_)_2_]. The main disadvantages of salt-containing aqueous, aqueous-organic, and organic solutions of fibroin are the long preparation time (aqueous solutions of fibroin should be dialyzed for several days). It should be noted that the concentrations of salts in such solutions reach the saturation limit. It was reported [23] that the efficiency of aqueous salt systems depends on the salt concentration and increases in the following order: for anions, sulfate < citrate < tartrate < acetate < chloride < nitrite < bromide < iodide < thiocyanate < dichloroacetate; for cations, Ca^2+^ < Sr^2+^ < Ba^2+^ < Li^+^ < Zn^2+^. A 75 : 25 (weight ratio) mixture of Ca(NO)_2_ · 4H_2_O and absolute methanol was used earlier for dissolving *Bombyx mori* silk [24, 25] as it has the strongest dissolving capacity for the SF.

Some solvent systems containing LiBr, LiCNS, and Ca(CNS)_2_ are unfavorable because LiBr, LiCNS, and Ca(CNS)_2_ are classified as toxic chemicals. Hence, in this study two solvent systems CaCl_2_ : EtOH : H_2_O (1 : 2 : 8 mole ratio) and Ca(NO)_2_ · 4H_2_O were utilized for SF processing.

Since purification of SF by dialysis usually takes 3-4 days and is applicable only for small batches of SF solution, we attempted to develop a scalable process using Sephadex G-25 media as described in the literature [19]. Effective chromatographic separation of SF from salt in solution was demonstrated by the data shown in Figure 1. Both UV absorbance and conductivity measurements for detecting SF and CaCl_2_, respectively, were quick and effective techniques for differentiating between the two solution components. Analysis of solids content upon drying provided an effective means of quantifying sample mass in each fraction collected, when combined with the latter experimental techniques to determine the composition of each fraction. High yield (95% recovery of SF protein) and high productivity (>98% salt removal in <2 hrs.) shown herein for Sephadex column chromatography provide a promising alternative to conventional SF purification by dialysis. Purification of SF solutions by Sephadex G-25 column chromatography could be an effective and industrially scalable chemical process. However, further optimization and analysis need to be performed for utilizing SF in pharmaceutical development. In addition, Sephadex media can be flushed and reused, thereby reducing development costs associated with purification of SF solutions.

### 4.2. Design of SF-Based Controlled Release Systems

SF is dominated in composition by the amino acids glycine, alanine and serine which tend to form antiparallel *β*-sheets or crystals through hydrogen bonding and hydrophobic interactions. Upon gelation a random coil structure of the SF transformed into *β*-sheet structure. Several factors affect the gelation of the SF aqueous solutions. Many factors such as temperature, SF concentration, shear force, metallic ions, Ca^2+^, pH, treatment with low dielectric constant solvents and poly(ethylene oxide) [21, 26] are thought to affect the conformation transition. With increase in SF content and temperature, physical cross-linking among SF chains formed more easily. Ca^2+^ ions accelerated these interactions through the hydrophilic blocks at the chain ends [27].

 It is well known and reported in the literature [14] that the addition of methanol to SF induces aggregation (dehydration), which drives the structural transition from random coil to **β**-sheet. It was demonstrated [28, 29] that upon methanol-induced crystallization, the SF **β**-sheet network stabilizes SF/gelatin hydrogels at elevated temperatures. The transition of regenerated SF films from random coil to **β**-sheet has been reported [30] after treatment with methanol, ethanol, and 2-propanol. It was also demonstrated [31] that the rate of gelation of SF was dependent upon glycerol content and/or SF content and addition of glycerol to the SF solution accelerated this rate.

In our research, we investigated the effect of dehydrating solvents (methanol, ethanol, isopropyl alcohol, and glycerin) on formation of **β**-sheets in SF/gelatin blends and demonstrated that the treatment with glycerin is also effective for the transformation of silk I to II which is in agreement with the literature data [32]. The presence of glycerin in the matrix can trigger **β**-sheet induction as seen from Table 3 at the ratio of SF/gelatin ~1 : 1. Since the **β**-sheet formation did not occur in experiments with SF-to-gelatin ratio of 1 : 3, it is suggested that the ratio of SF to gelatin is also critical for the *β*-sheet formation. In the presence of glycerin, for the SF/gelatin 1 : 1 blend, untreated films exhibit the absorption bands characteristic of the **β**-sheet structure. In this case the FTIR spectra possess strong absorption bands at 1625.9 cm^−1^–1621.8 cm^−1^ and 1536 cm^−1^–1531.9 cm^−1^, corresponding to the amide I and amide II peaks, respectively.

**Figure 8:**
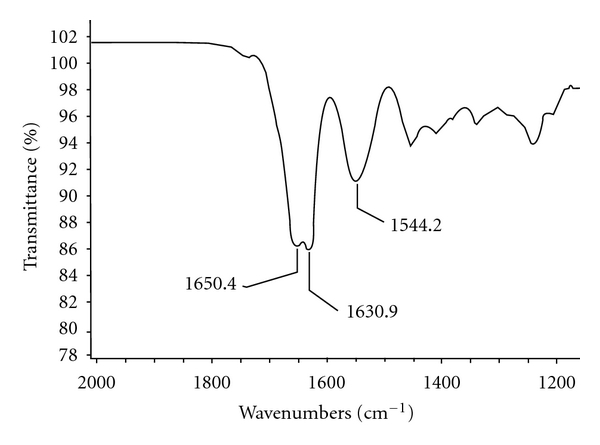
FTIR spectra of SF/gelatin/glycerin matrix.

 The induced crystallization of SF-containing films had an impact on the release profile of the model drug naproxen sodium as evidenced by dissolution studies performed on naproxen-sodium-loaded films. It was shown that no burst effect was observed for matrix containing SF : gelatin : glycerin in the ratio of 1 : 3 : 3 compared to films containing gelatin alone or silk : gelatin (1 : 1.5) only which released almost 80% of the drug within the first 15 minutes (Figure 2). The influence of glycerin-induced SF/gelatin crystallization on structure and properties was ascertained by dissolution studies of SF containing controlled release matrixes (Figure 2). The *β*-sheet content in the SF matrixes was assessed by FTIR and illustrated in Figure 8. Two maxima on spectra reflect the characteristic bands of noncrystallized biopolymer (gelatin) in the matrix and crystallized SF. The amide I peak, which reflects the stretching of C=O group along the SF backbone, is shifted from 1655 to 1630 cm^−1^, while the gelatin exhibit, the absorption band at 1654 cm^−1^ (amide I).

Release behavior of the model drug at different loading from spray-dried microparticles was studied using 3-stage dissolution testing conditions. In our study it was observed that the release profile was not dependant on the naproxen-to-SF ratios in the range of 3 : 1 to 1 : 1 or treatment with dehydrating solvent (ethanol) demonstrating that spray-drying method accelerated the transition from random coil to the *β*-sheet structure of microparticles, which is in agreement with the literature data [19].

Our data obtained from naproxen-loaded, spray-dried microparticles, matrices, and films demonstrated a promising approach for creating a new platform for controlled drug delivery.

## 5. Conclusions

It has been demonstrated that the conformational transition of SF from random coil to **β**-sheet in blends with gelatin obtained by spray-drying or induced by solvents could be used to generate a porous matrix. The development of SF-containing blends in which SF is crystallized yields drug delivery system allowing for controlled release of the drug. Further studies will be performed on SF-containing matrixes and microparticles to explore feasibility for delivering different classes of drugs, in particular macromolecular drugs for site-specific delivery.

## Figures and Tables

**Table 1:** SF Processing Conditions.

Silk type	Degumming solution	Dissolving solution
Silk worm cocoons—Bombyx mori	0.25% Na_2_CO_3_	9.0 M LiBr
	0.25% Na_2_SO_4_	Ca(NO_3_)_2_ 4H_2_O : MeOH (75 : 25) w/w
Grade 5A Raw Silk—Bombyx mori	0.25% Na_2_CO_3_	Ca(NO_3_)_2_ 4H_2_O : MeOH (75 : 25) w/w
	0.25% C_12_H_25_O_4_SNa	CaCl_2_ : EtOH : H_2_O (1 : 2 : 8) mole ratio
Silk fibroin powder	Not required	CaCl_2_ : EtOH : H_2_O (1 : 2 : 8) mole ratio

**Table 2:** FT-IR analysis of partially hydrolyzed SF in blends with gelatin.

	Amide I (cm^−1^)	Amide II (cm^−1^)
Pure SF Films		
2 kDa SF	1620	—
14 kDa SF	1624	1520
100 kDa SF	1625	1519

SF/gelatin blends		
2 kDa SF (1.8%)	1636	1542
12 kDa SF (0.9%)	1632	1540
100 kDa SF (1.4%)	1625	1538

**Table 3:** Effect of different solvents on *β*-sheet formation.

SF/gelatin composition	Solvent	Absorption bands (cm^−1^)	
		Amide I	Amide II
SF/G (1 : 3) without glycerin	Untreated	1646.3	1540.1
	MeOH	1630.0	1540.1
	EtOH	1644.2	1537.7
	IPA	1630.0	1536.0
SF/G (1 : 3) with glycerin	Untreated	1650.4	1544.2–1548.2
	MeOH	1630.0	1536.0
	EtOH	1638.1	1536.0
	IPA	1630.0	1536.0
SF/G (1 : 1) with glycerin	Untreated	1625.9	1536.0–1531.9
	MeOH	1625.9	1527.8
	EtOH	1621.8	1527.8
	IPA	1621.8	1536.0

**Table 4:** Composition of naproxen sodium experimental samples.

Components	Silk : gel : glycerin (1 : 3 : 3) Matrix	Silk fibroin film	Control film
Naproxen sodium (mg)	102.4	250.0	250.0
Gelatin (g)	0.575	0.150	0.250
Silk fibroin (g)	0.192	0.160	0.000
Glycerin (g)	0.575	0.075	0.250

**Table 5:** SF : NS spray-dried powder blend compositions.

Composition	Ratio (w/w)
3% SF	—
3% SF : 5% EtOH	2 : 1
3% SF : 1% NS	1 : 1
3% SF : 1% NS	1.5 : 1
3% SF : 1% NS	2 : 1
3% SF : 1% NS	3 : 1
3% SF : 1% NS (5% EtOH)	2 : 1

**Table 6:** FT-IR comparison of amide I, II, and III shifts in SF powder blends exposed to 76% humidity.

Composition	Amide I (cm^−1^)	Amide II (cm^−1^)	Amide III (cm^−1^)
SF : NS (1 : 1) 76% humidity	1658.2/1629.5	1548.3/1515.7	1264.0/1230.5
SF : NS (1.5 : 1) 76% humidity	1656.6/1629.9	1538.4/1515.9	1262.6/1228.7
SF : NS (1 : 1) no humidity	1658.3	1546.6	1230.0
